# Evaluation of a novel ^177^Lu-labelled therapeutic Affibody molecule with a deimmunized ABD domain and improved biodistribution profile

**DOI:** 10.1007/s00259-024-06840-5

**Published:** 2024-07-15

**Authors:** Yongsheng Liu, Maryam Oroujeni, Yunqi Liao, Anzhelika Vorobyeva, Vitalina Bodenko, Anna Orlova, Mark Konijnenberg, Matilda Carlqvist, Elisabet Wahlberg, Annika Loftenius, Fredrik Y Frejd, Vladimir Tolmachev

**Affiliations:** 1 Department of Immunology, Genetics and Pathology, Uppsala University, Uppsala, 751 85 Sweden; 2 Affibody AB, Solna, 171 65 Sweden; 3 Department of Medicinal Chemistry, Uppsala University, Uppsala, 751 23 Sweden; 4 Department of Radiology & Nuclear Medicine, Erasmus MC, Rotterdam, The Netherlands

**Keywords:** Affibody molecules, Albumin binding domain, Lutetium-177, Therapy

## Abstract

**Purpose:**

Fusion of Affibody molecules with an albumin-binding domain (ABD) provides targeting agents, which are suitable for radionuclide therapy. To facilitate clinical translation, the low immunogenic potential of such constructs with targeting properties conserved is required.

**Methods:**

The HER2-targeting Affibody molecule ZHER2:2891 was fused with a deimmunized ABD variant and DOTA was conjugated to a unique C-terminal cysteine. The novel construct, PEP49989, was labelled with ^177^Lu. Affinity, specificity, and in vivo targeting properties of [^177^Lu]Lu-PEP49989 were characterised. Experimental therapy in mice with human HER2-expressing xenografts was evaluated.

**Results:**

The maximum molar activity of 52 GBq/µmol [^177^Lu]Lu-PEP49989 was obtained. [^177^Lu]Lu-PEP49989 bound specifically to HER2-expressing cells in vitro and in vivo. The HER2 binding affinity of [^177^Lu]Lu-PEP49989 was similar to the affinity of [^177^Lu]Lu-ABY-027 containing the parental ABD035 variant. The renal uptake of [^177^Lu]Lu-PEP49989 was 1.4-fold higher, but hepatic and splenic uptake was 1.7-2-fold lower than the uptake of [^177^Lu]Lu-ABY-027. The median survival of xenograft-bearing mice treated with 21 MBq [^177^Lu]Lu-PEP49989 (> 90 days) was significantly longer than the survival of mice treated with vehicle (38 days) or trastuzumab (45 days). Treatment using a combination of [^177^Lu]Lu-PEP49989 and trastuzumab increased the number of complete tumour remissions. The renal and hepatic toxicity was minimal to mild.

**Conclusion:**

In preclinical studies, [^177^Lu]Lu-PEP49989 demonstrated favourable biodistribution and a strong antitumour effect, which was further enhanced by co-treatment with trastuzumab.

**Supplementary Information:**

The online version contains supplementary material available at 10.1007/s00259-024-06840-5.

## Introduction

The impressive development in radionuclide theranostics is associated with the use of small-size targeting moieties, such as inhibitors of prostate-specific membrane antigen or fibroblast activation protein, meta-iodobenzylguanidine derivatives, and targeting peptides [1–3]. These agents demonstrate a pronounced anti-tumour effect not only in hematologic malignancies but also in solid cancers. On the other hand, the repertoire of such targeting agents is so far limited. A possible solution for rapid development of targeting proteins is the use of engineered scaffold proteins (ESPs), small polypeptides that contain a firm and stable frame in combination with variable amino acids [4]. The structure of ESP permits the selection of high-affinity binders using combinatorial methodology. ESPs combine such advantages of short peptides, as fast target tissue accumulation, deeper and faster tissue penetration, controlled chemical modification, and production by chemical synthesis, with the advantages of monoclonal antibodies such as high affinity and stability to proteases in vivo [3, 5]. ESPs Affibody molecules have been successfully evaluated for radionuclide imaging [6, 7]. The most impressive progress was achieved with derivatives of the Affibody molecule ZHER2:342, which binds to human epidermal growth factor receptor type 2 (HER2) with an affinity of 22 pM [8]. HER2 is overexpressed in a variety of carcinomas [9] and is used as a target for therapy of breast and gastric cancers. Clinical studies of HER2 imaging agents based on derivatives of ZHER2:342 demonstrated specific targeting and efficient imaging of HER2-expressing lesions [10–15]. Importantly, clear tumour visualization was achieved within 2–3 hours after injection because the unbound tracer was rapidly cleared via kidneys and did not contribute to background. On the other hand, the cleared tracer was reabsorbed in renal proximal tubule. The renal uptake exceeded by far the uptake in tumours, which makes radionuclide therapy challenging. Commonly used methods for reduction of the renal uptake of radiolabelled peptides, e.g. injections of cationic amino acids or Gelofusine, were inefficient in this case [16]. To enable Affibody molecule-mediated radionuclide therapy, several techniques were evaluated in preclinical models, including pretargeting [17, 18] and the use of non-residualizing labels [19, 20]. A successful approach for the reduction of renal uptake of Affibody molecules was a fusion with an albumin-binding domain (ABD) [21]. Binding to albumin in blood reduces glomerular filtration of such constructs. The first variant containing a dimeric first-generation Affibody molecule ZHER2:342 ([^177^Lu]Lu-CHX-A’’-DTPA-ABD-(ZHER2:342)_2_) demonstrated clear anti-tumour effect in preclinical radionuclide therapy [21]. Optimization of the fusion architecture resulted in the development of a novel targeting agent [^177^Lu]Lu-ABY-027 [22], containing a monomeric second-generation Affibody molecule ZHER2:2891 as a HER2-binding moiety and a DOTA chelator conjugated site-specifically at the C-terminus of a high-affinity derivative of ABD, ABD035 [23]. The renal uptake of [^177^Lu]Lu-ABY-027 was four-fold reduced compared to the uptake of [^177^Lu]Lu-CHX-A’’-DTPA-ABD-(ZHER2:342)_2_ [22]. Experimental therapy using [^177^Lu]Lu-ABY-027 demonstrated a significant increase in the survival of mice with implanted human HER2-expressing tumours [24]. Attempts to further improve the design of ABD-fused HER2 targeting Affibody molecules, e.g. by changing the conjugation site of the DOTA chelator [25, 26] resulted in a significant increase of renal uptake. Thus, the overall design of ABY-027 remains preferable.

In radioimmunotherapy, formation of neutralizing antibodies was one major issue for treatment using murine antibodies [27]. The use of chimeric antibodies alleviated this problem but formation of human anti-chimeric antibodies was still found in 13–60% patients during clinical trials [28, 29]. Even the use of humanized antibodies is associated with formation of anti-human antibodies in 12–14% of patients undergoing radioimmunotherapy [30]. To prevent this in the case of ABD-fused Affibody molecules, a deimmunized variant of the affinity matured ABD was developed as previously described in Zurdo et al. [31]. In short, the original ABD domain was isolated from streptococcal protein G and has undergone affinity maturations using phage display, which resulted in the ABD035 variant. ABD035 was found to still contain a T-cell epitopes and was therefore subjected to de-immunization via protein engineering. By using the Epibase *in silico immunogenicity program*, several new ABD variants were selected based on reduced immunogenicity potential. The most promising ABD variants were expressed in *E.coli*, purified and characterized for retained affinity and stability. Three variants were then assessed for their ability to activate CD4 + T-cells by using peripheral blood mononuclear cells (PBMC) from 52 healthy blood donors. Less than 4% of the donors responded to the ABD* variant.

**Fig. 1:**
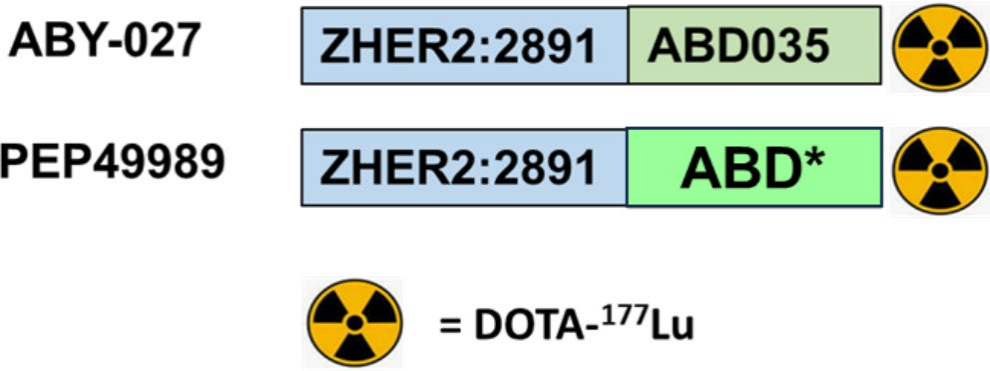
General architecture of tested constructs. ABD* refer to the deimmunized ABD variant [31]

The goal of the current study was to evaluate how the use of this ABD* variant influences the targeting properties of Affibody molecule-based constructs for radionuclide therapy. Therefore, we fused the C-terminus of the ZHER2:2891 Affibody molecule with the deimmunized ABD* containing a C-terminal cysteine, which was coupled to a DOTA chelator. This agent was designated PEP49989. Targeting properties of [^177^Lu]Lu-labelled PEP49989 and ABY-027 were directly compared. Since combination treatment is considered as a promising approach for therapy of metastatic cancer [1, 3, 32], we compared [^177^Lu]Lu-PEP49989-mediated radionuclide therapy with and without trastuzumab in tumour-bearing animals Fig. 1.

## Materials and methods

### Protein production and labelling chemistry

Information concerning protein production, purification and characterisation of targeting proteins is provided in the Supplementary material (Supplementary Figs. 1 and 2). For the labelling of ABY-027 and PEP49989 with ^177^Lu, an aliquot of a protein in 0.2 M sodium acetate, pH 6.0, was mixed with [^177^Lu]LuCl_3_ (no-carrier-added, Curium Pharma Stockholm, Sweden) solution to reach a molar activity of 30–50 MBq/nmol. The buffer volume was four-fold bigger than the volume of radionuclide solution. After incubation at 65 °C for 30 min, the radiochemical yield was determined by instant thin layer chromatography (iTLC, Agilent Technologies, Folsom, CA, USA) eluted with 0.2 M of citric acid, pH 2.0. After purification, the purity was evaluated using iTLC. To validate iTLC, radio-HPLC was performed (See Supplementary Fig. 3).

To evaluate label stability, aliquots of the freshly radiolabelled conjugates were incubated with 500-fold molar excess of EDTA at 37 °C for 60 min. Incubation was also performed in PBS as a control. Samples were evaluated in triplicate.

To test the most appropriate storage and shipment conditions of labelled protein PEP49989, three approaches were tested: dilution with PBS (to reduce the absorbed dose and minimize the radiolysis), freezing at -20 ^o^C, or adding 6 mM sodium ascorbate and storage at ambient temperature. [^177^Lu]Lu-PEP49989 was purified using size-exclusion chromatography to obtain a radiochemical purity 99.4%. Two aliquots (150 µL each) were stored at ambient temperature. Eight aliquots (30 µL each) were frozen at -20^o^C. To two aliquots (150 µL each), 20 µl sodium ascorbate (10 µg/µL) were added and samples were stored at ambient temperature. The percentage of protein-bound activity was measured using iTLC 1, 2, 20, and 96 h after incubation start.

### In vitro studies

The binding specificity of radiolabelled compounds to HER2-expressing cells was evaluated by in vitro blocking test as described in [25] using SKOV3 and BT474 HER2-expressing cells (both from American Type Culture Collection). Experiments were performed in triplicate. To evaluate the impact of human serum albumin (HSA) on binding specificity, an additional set of experiments was performed in a medium containing 100 nM of HSA. To further confirm that the binding of [^177^Lu]Lu-PEP49989 is HER2-mediated, its binding to HER2-negative cell lines was evaluated in an additional set of experiments (See Supplementary Materials).

The affinity of [^177^Lu]Lu-ABY-027 and [^177^Lu]Lu-PEP49989 binding to SKOV3 cells was measured using LigandTracer (Ridgeview Instruments AB, Vänge, Sweden), as described in [26] using SKOV3 cells. The data were analysed by InteractionMap software (Ridgeview Instruments AB, Uppsala, Sweden) to calculate the dissociation constant at equilibrium (K_D_).

Cellular processing of [^177^Lu]Lu-ABY-027 and [^177^Lu]Lu-PEP49989 by HER2-expressing SKOV3 and BT474 cell lines in the presence of HSA was compared using the acid wash method as described in [19].

### In vivo studies

The animal experiments were performed in accordance with national legislation on laboratory animal protection, and the study was approved by the local Ethics Committee for Animal Research in Uppsala (permit 5.8.18–11,931/2020, 28 August 2020). Four mice per data point were used in biodistribution experiments. HER2-positive and HER2-negative xenografts were established by subcutaneous injection of approximately 10^7^ SKOV3 cells or Ramos cells, respectively, in the hind legs of female BALB/C nu/nu mice. Mice were injected intravenously with [^177^Lu]Lu-labelled conjugates in 100 µL of 1% BSA in PBS. The injected activity was 270 kBq/mouse, and the injected protein mass was adjusted to 10 µg/mouse using non-labelled compounds.

To compare the targeting properties of [^177^Lu]Lu-labelled ABY-027 and [^177^Lu]Lu-PEP49989, biodistribution was evaluated in mice bearing SKOV3 xenografts 48 and 264 h after injection. The average tumour weight was 0.67 ± 0.31 g.

Biodistribution of [^177^Lu]Lu-PEP49989 was measured at 4, 24, 48, 72, 168 and 336 h to obtain the data for dosimetry evaluation. To confirm HER2-specificity of [^177^Lu]Lu-PEP49989 uptake in tumours, one group of mice was injected with 700 µg/mouse unlabelled PEP49989 24 h before injection of [^177^Lu]Lu-PEP49989. As another control, mice with HER2-negative Ramos lymphoma xenografts were injected with [^177^Lu]Lu-PEP49989. The biodistribution of [^177^Lu]Lu-PEP49989 in these groups was measured 48 h after injection. The average animal weight was 18.1 ± 1.6 g at the time of the experiment. The average SKOV3 tumour weight was 0.4 ± 0.2 g. The average Ramos tumour weight was 0.8 ± 0.4 g.

Based on the biodistribution data, dosimetry for mice was calculated as described in [33]. The injected activity for a preclinical therapy was selected based on data concerning the growth rate of SKOV-3 xenografts (obtained from previous animal studies) and data for the radiosensitivity of SKOV-3 cells obtained in radiobiology studies.

In vivo imaging was performed to confirm the biodistribution data. One mouse with SKOV3 xenograft and one mouse with Ramos xenograft were injected with 10.5 MBq (10 µg) of [^177^Lu]Lu-PEP49989. The imaging was performed 48 h p.i. using nanoScan SPECT/CT (Mediso Medical Imaging Systems, Budapest, Hungary). The data were reconstructed using Tera-Tomo™ 3D SPECT Software, as described in [26].

### Experimental radionuclide therapy

Female Balb/c nu/nu mice were subcutaneously implanted with SKOV3 cells (approximately 10^7^ cells per animal) on the abdomen area. The mice were randomized into five groups of seven to nine mice each and the treatment of animals was initiated six days after implantation. By the time of injection, the average tumour volume was 0.16 ± 0.04 cm^3^ and the average mouse weight was 18.2 ± 0.8 g.

A schematic illustration of the timing of the interventions in the treatment experiment is presented in Supplementary Fig. 9. In one group, mice were injected i.v. with 10 µg (21 MBq) of [^177^Lu]Lu-PEP49989. In another group, mice received two injections of [^177^Lu]Lu-PEP49989, the first on day 1 and the second on day 29. Animals in the control group received vehicle, 1% BSA in PBS, alone. To evaluate the effect of trastuzumab, one group of mice were weekly s.c. injected with trastuzumab (Roche Registration GmbH, Germany) with a loading dose of 4 mg/kg for the 1st week and thereafter with 2 mg/kg for 5 consecutive weeks. To evaluate the effect of combination therapy of trastuzumab and [^177^Lu]Lu-PEP49989, a group of mice was injected i.v. with 10 µg (21 MBq) of [^177^Lu]Lu-PEP49989 and trastuzumab with the same dose as the group injected with trastuzumab alone. Mice were weighted twice a week and tumours were measured using a calliper. The tumour volume (V) was then calculated using the formula V = 1/2 × (length × width^2^). Mice were euthanized if their tumour volume exceeded 1000 mm^3^, if the tumours became ulcerated, or if the animals lost over 10% of their weight within 1 week, or more than 15% since the study began. After euthanasia, kidneys, livers and tumours were collected and examined by an experienced animal pathologist at BioVet AB veterinary medicine lab (Sollentuna, Sweden) to detect any histopathologic changes.

## Results

### Protein production and characterisation

Results from kinetic characterisation of targeting constructs was obtained by Surface Plasmon Resonance (Table 1). Identity and purity of novel constructs were confirmed using LC-MS (Table 1 and Supplementary Fig. 1). Circular dichroism analysis showed similar spectra, with two local inflexion points at 208 and 221 nm, indicating high alpha-helical content. The proteins refolded with high fidelity after heating to 90 ^o^C (Supplementary Fig. 2).

**Table 1:** Characterization of ABD-fused constructs. Affinity data from Surface Plasmon resonance measurements

Targeting agent	Mass calculated (Da)	Mass found (Da)	Purity (%)	Affinity to HER2 (M)	Affinity to HSA (M)	Affinity to MSA (M)
PEP49989	12,460	12,461	> 95	3.4 × 10^− 10^	2.5 × 10^− 11^	3.6 × 10^− 10^
ABY-027	12,646	12,650	99	5.7 × 10^− 11^	5.3 × 10^− 13^	3.6 × 10^− 11^

### Labelling

The maximum radiochemical yield was achieved after 30 min. Further increase of incubation time did not increase yield. The labelling resulted in a yield of over 97% at a molar activity of 52 GBq/µmol. After incubation with a 500-fold molar excess of EDTA for 1 h, the radiochemical purity of [^177^Lu]Lu-PEP49989 was 99 ± 0%, which was not different from purity after incubation with PBS.

Data concerning stability of [^177^Lu]Lu-PEP49989 at different storage conditions are presented in Table 2. The data show that the addition of ascorbate as a radical scavenger provides the best shelf-life of [^177^Lu]Lu-PEP49989 and enables shipment at ambient temperature of the pre-labelled product to any point globally.

**Table 2:** Stability of [^177^Lu]Lu-PEP49989 at different storage conditions analysed by iTLC. The initial radiochemical purity of the labelled protein was 99.4% after purification on NAP-5

	Percentage of protein-associated activity at different time points			
Storage conditions	1 h	2 h	20 h	96 h
Dilution in PBS at RT^a^	98.85 ± 0.35	97.9 ± 1.0	85.9 ± 0.3	33.4 ± 2.2
Ascorbate at RT^a^	99.25 ± 0.05	98.8 ± 0.3	97.7 ± 0.1	95.6 ± 1.1
Frozen at -20 ^o^C	99 ± 0.3	98.4 ± 0	93.75 ± 0.75	70 ± 22

^a^ RT room temperature (ambient)

### In vitro studies

**Fig. 2:**
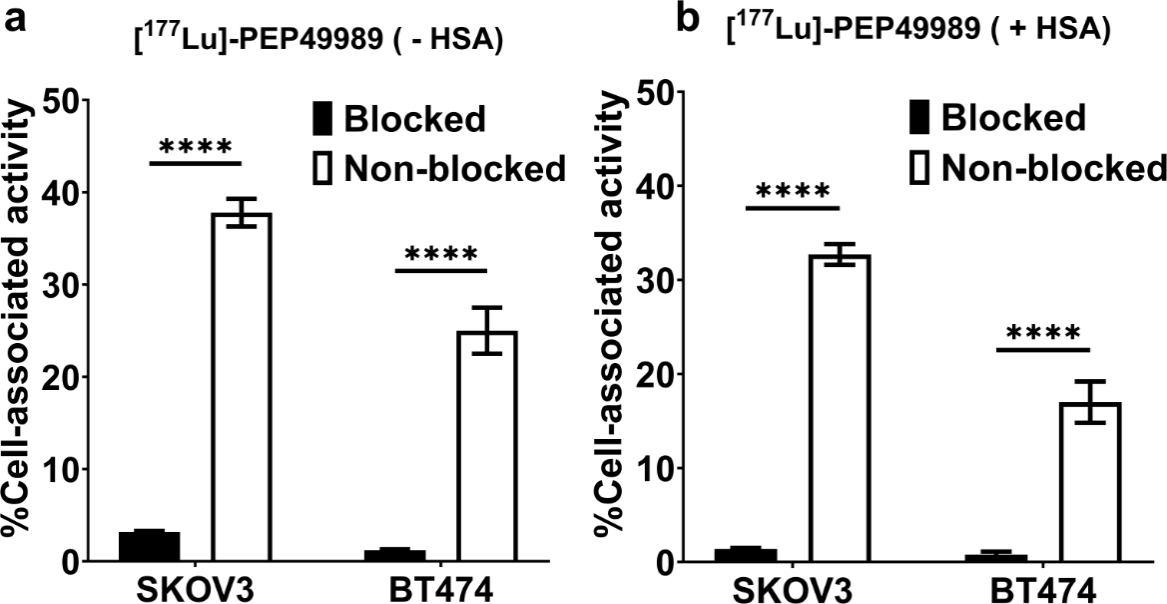
In vitro specificity of [^177^Lu]Lu-PEP49989 binding to HER2-expressing SKOV3 and BT474 cells in the absence (**a**) and presence (**b**) of HSA. Blocked cells were treated with 1000-fold excess of an anti-HER2 Affibody conjugate. The data are presented as an average value from 3 samples ± SD. Asterisks (*) mark a highly significant difference (*p* < 0.0005, unpaired t-test)

The results of the in vitro binding specificity tests of [^177^Lu]Lu- PEP49989 are shown in Fig. 2. Binding of [^177^Lu]Lu-labelled conjugates to HER2-expressing SKOV3 and BT474 cells was significantly lower (*p* < 0.001) after saturation of HER2 receptors in blocked groups both in the presence and absence of HSA. Furthermore, the binding to HER2-negative cells was much lower than to positive (Supplementary Fig. 4).

The affinities of [^177^Lu]Lu-PEP49989 and [^177^Lu]Lu-ABY-027 binding to living HER2-expressing SKOV3 cells in the presence of HSA were evaluated head-to-head by InteractionMap analysis of the LigandTracer sensorgrams. The data are presented in Table 3 and in Supplementary Fig. 5.

**Table 3:** InteractionMap evaluation of the affinity of [^177^Lu]LuPEP49989 and [^177^Lu]Lu-ABY-027 binding to HER2-expressing SKOV3 cells in the presence of HSA

	K_D1_ (pM)	Weight (%)	K_D2_ (nM)	Weight (%)
PEP49989	327 ± 12	61 ± 9	11.5 ± 1.7	23 ± 8
ABY-027	341 ± 4	61 ± 1	11.7 ± 4.5	23 ± 1

The InteractionMap analysis for both labelled Affibody molecules showed a one-to-two binding with high association and low dissociation rates, resulting in predominant affinity in sub-nanomolar range. Affinities of [^177^Lu]Lu-ABY-027 and [^177^Lu]Lu-PEP49989 were approximately equal to each other (Table 3).

**Fig. 3:**
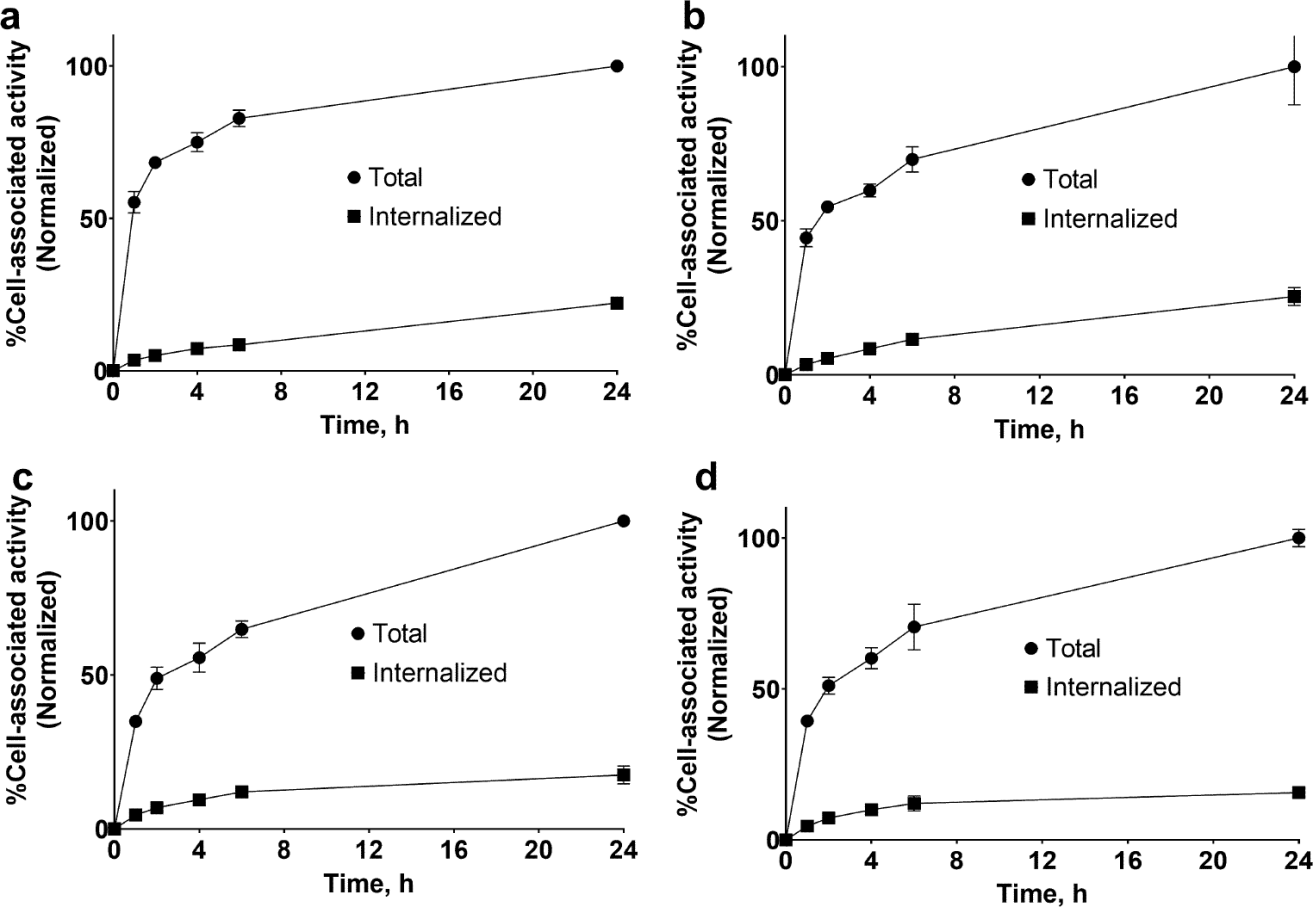
Comparison of binding and cellular processing of [^177^Lu]Lu-PEP49989 (**a**, **c**) and [^177^Lu]Lu-ABY-027 (**b**, **d**) by SKOV3 (**a**, **b**) and BT474 (**c**, **d**) cells. Data are presented as an average (*n* = 3) value ± SD

The data concerning the comparison of cellular processing of [^177^Lu]Lu-PEP49989 and [^177^Lu]Lu-ABY-027 by cancer cells in the presence of HSA are shown in Fig. 3. The cellular processing pattern was similar for both targeting agents. The binding was rapid during the first 2 h of incubation. The internalization in both cell lines was slow, less than 20% in 24 h.

### In vivo studies

**Fig. 4:**
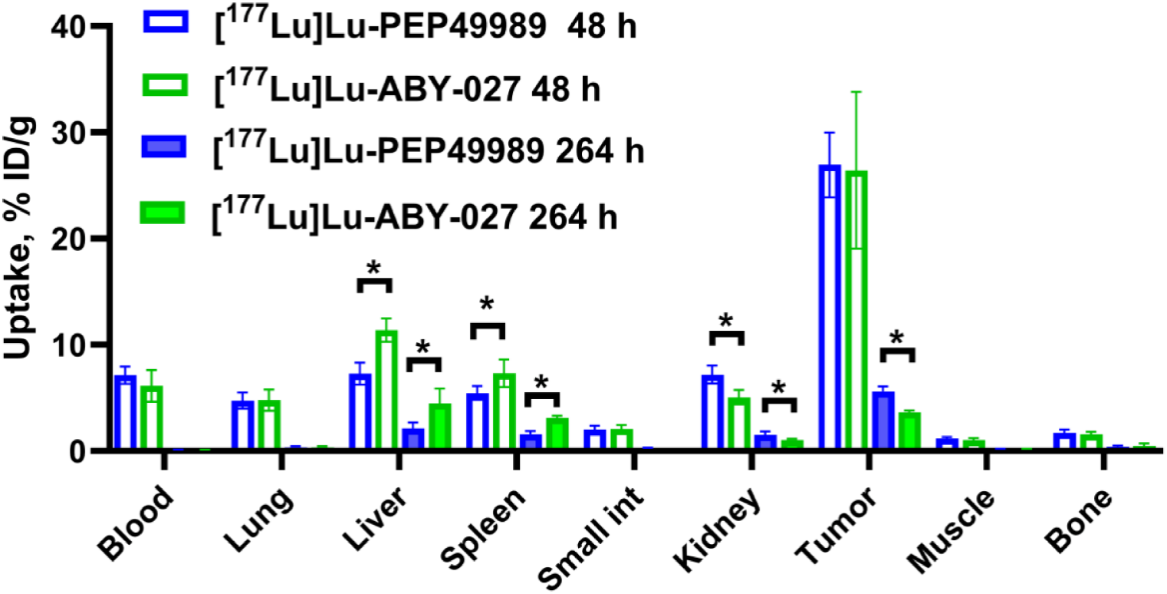
Biodistribution of [^177^Lu]Lu-PEP49989 and [^177^Lu]Lu-ABY-027 in tumour-bearing mice. Data are presented as an average (*n* = 4) value ± SD. Asterisks (*) mark a significant difference (*p* < 0.05, unpaired t-test)

Results of the comparison of biodistribution of [^177^Lu]Lu-labelled ABY-027 and PEP49989 in mice bearing SKOV3 xenografts are shown in Fig. 4, and detailed results of the statistical analysis are provided in Supplementary Figs. 6 and 7. Uptake of all constructs was similar (*p* > 0.05 in unpaired t-test) in a majority of organs at both time points. However, the kidney uptake of [^177^Lu]Lu-PEP49989 was persistently 1.4-fold (*p* < 0.05) higher than the uptake of [^177^Lu]Lu-ABY-027. On the opposite, the uptake of [^177^Lu]Lu-PEP49989 in the liver was significantly 1.7-2.0 fold (*p* < 0.05) lower than the uptake of [^177^Lu]Lu-ABY-027. The tumour uptake of [^177^Lu]Lu-PEP49989 was significantly (*p* < 0.05, ca. 1.5-fold) higher than the uptake of [^177^Lu]Lu-ABY-027 at 264 h p.i.

**Fig. 5:**
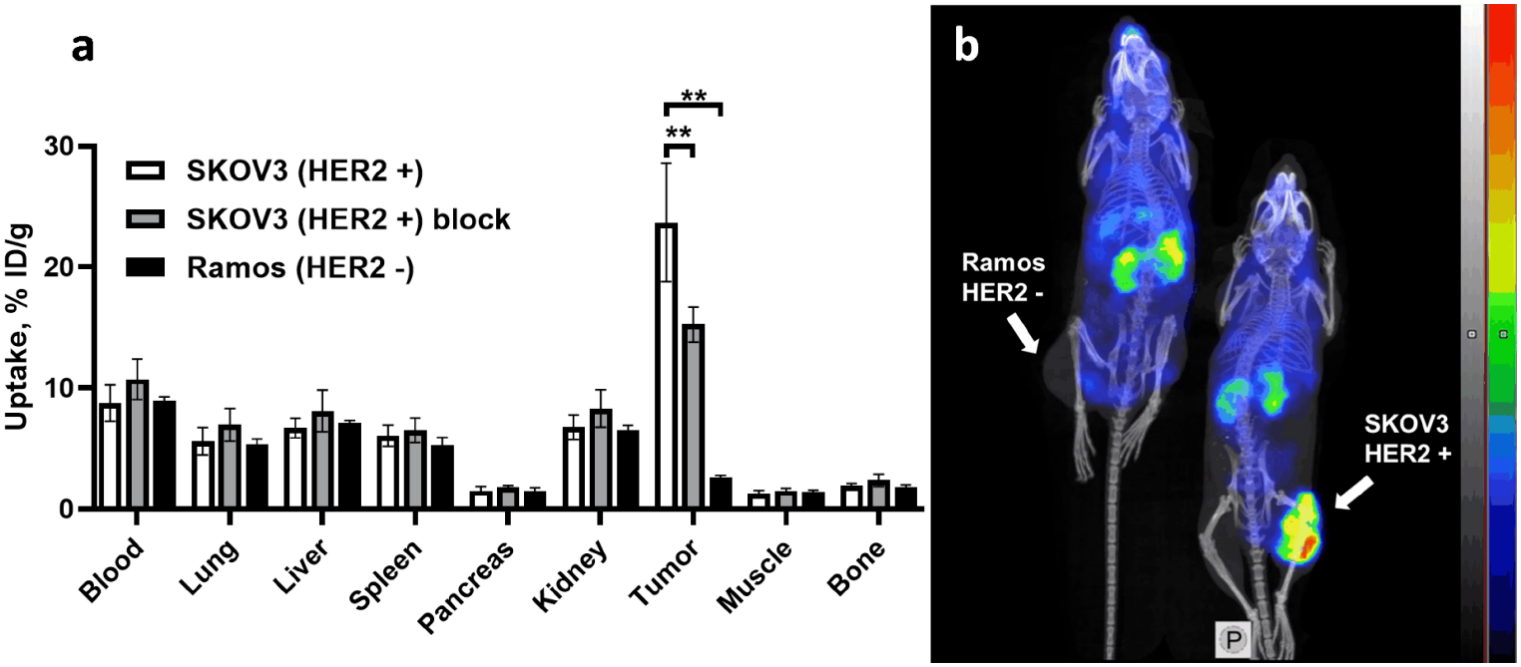
The HER2-specificity of [^177^Lu]Lu-PEP49989 accumulation in tumours. (**a**) Biodistribution in mice HER2-positive SKOV3 and HER2-negative Ramos xenografts. In the blocking group, HER2 in SKOV3 xenografts were saturated by pre-injection of 700 µg unlabelled PEP49989. Asterisks (**) mark a highly significant difference (*p* < 0.005, unpaired t-test). Results are presented as % ID/g ± SD (*n* = 4–8). (**b**) Imaging of [^177^Lu]Lu-PEP49989 in Balb/c nu/nu mice bearing SKOV3 and Ramos xenografts 48 h after injection. The scale is linear showing arbitrary units normalized to a maximum count rate

In vivo specificity test showed that the uptake of [^177^Lu]Lu-PEP49989 in HER2-negative Ramos xenografts was 9.5-fold (*p* < 0.0005) lower than that in HER2-expressing SKOV3 xenografts (Fig. 5a). Pre-injection of unlabelled PEP49989 resulted in significantly (*p* < 0.005) lower uptake in SKOV3 xenografts. The results were confirmed by imaging performed 48 h after injection of [^177^Lu]Lu-PEP49989 both in SKOV3 and Ramos xenografts (Fig. 5b).

**Fig. 6:**
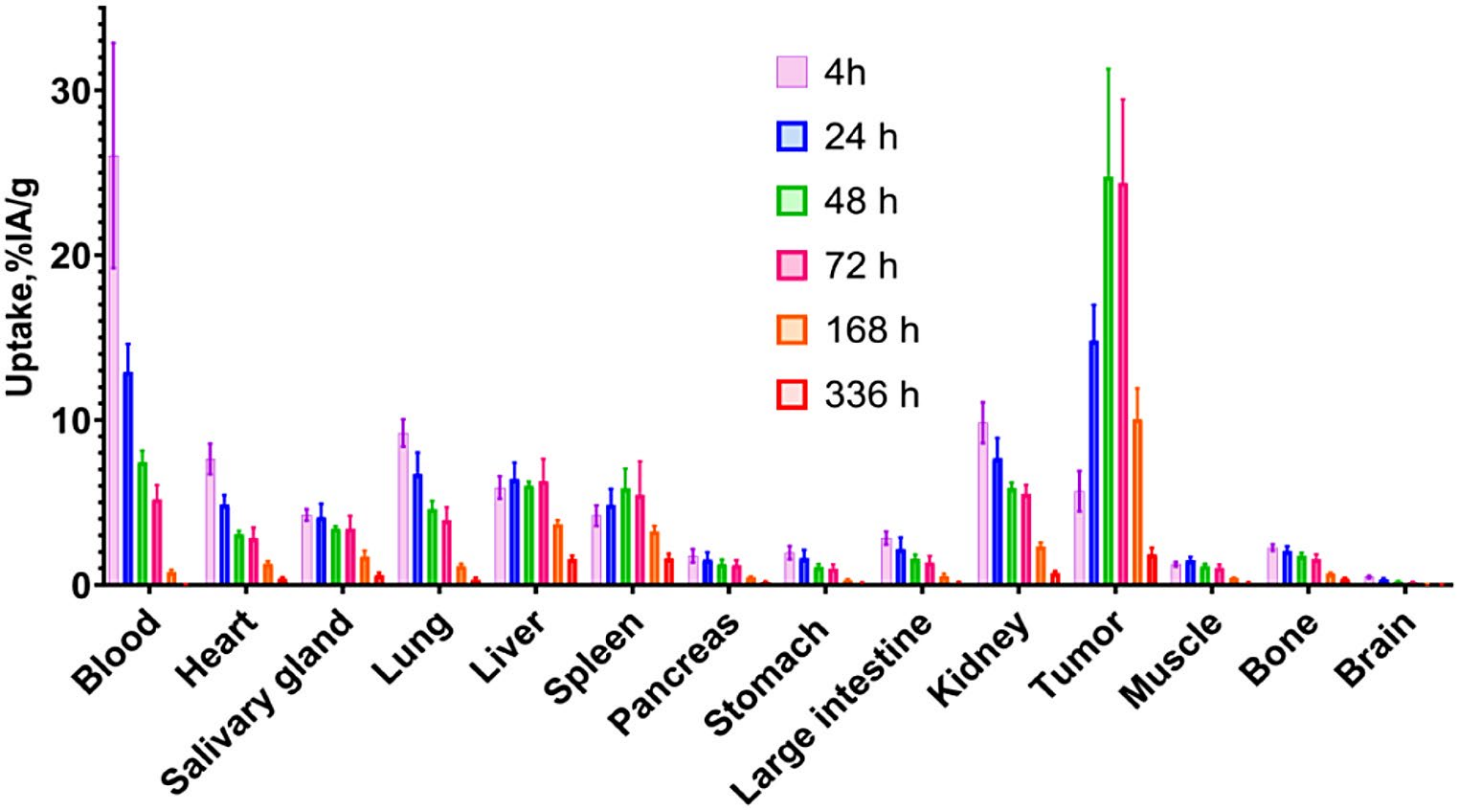
Biodistribution of [^177^Lu]Lu-PEP49989 in mice bearing SKOV3 xenografts. Data are presented as an average (*n* = 4) value ± SD

Biodistribution of [^177^Lu]Lu-PEP49989 in mice bearing SKOV3 xenografts is presented in Fig. 6. A slow clearance of [^177^Lu]Lu-PEP49989 from the blood was observed, with a biological half-life of 23 h. Clearance of [^177^Lu]Lu-PEP49989 from normal organs and tissues followed clearance from the blood. By 24 h, the tumour uptake was higher than the uptake in normal organs. The tumour uptake peaked between 48 and 72 h after injection, followed by a slow washout (T_1/2_ =39 h). At these time points, the tumour uptake was 4-fold higher than the uptake in the kidney.

Absorbed doses in mice, which were calculated based on the biodistribution, are provided in Supplementary Table 1. Based on these calculations, the injected activity, 21 MBq, was selected as providing the highest tumour control probability while keeping acceptable levels of absorbed dose to bone marrow (1.24 Gy), kidneys (10.1 Gy) and liver (10 Gy). An additional experiment (See Supplementary materials, Supplementary Fig. 14 and related text) demonstrated that exceeding this activity results in unacceptable weight loss and euthanasia of mice within 10 to 15 days after injection. These data suggest that 21 MBq can be considered as the maximum tolerated activity in mice.

### Experimental therapy study

**Fig. 7:**
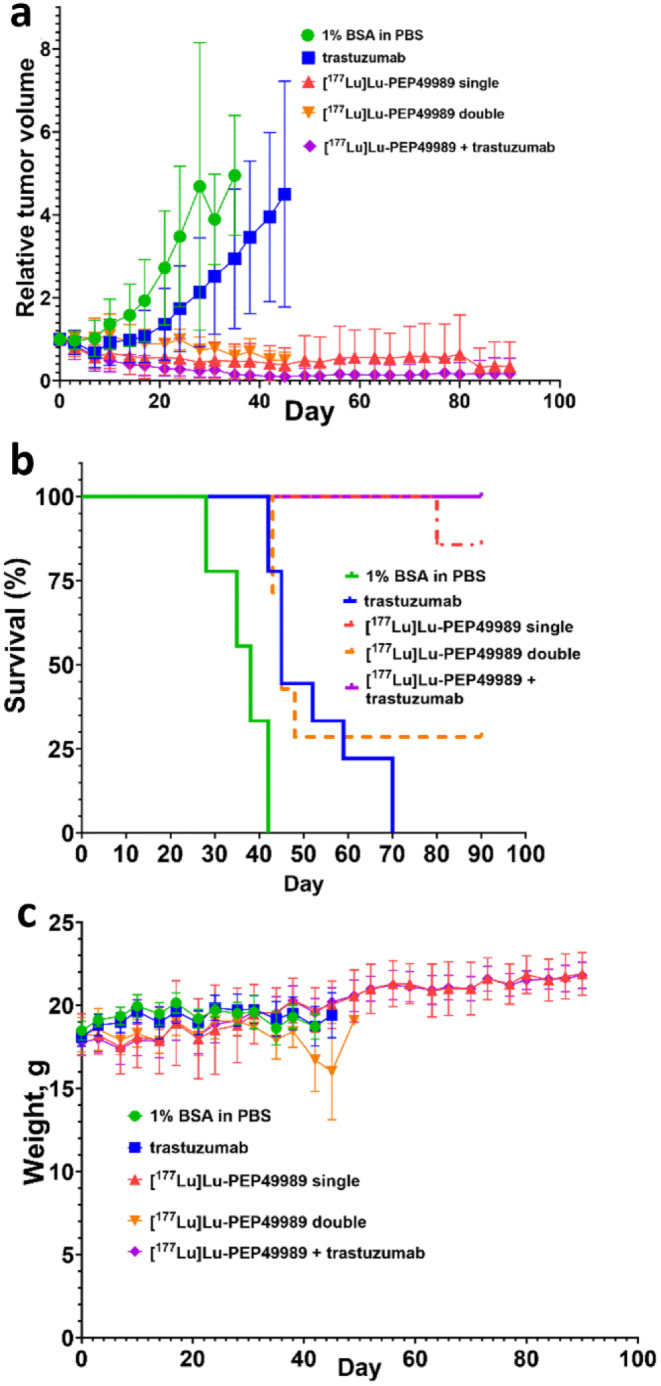
The therapeutic efficacy of [^177^Lu]Lu-PEP49989 in the SKOV3 model. (**a**) Tumour growth curves, (**b**) survival and (**c**) weight of the treated mice

The result of the experimental therapy is presented in Fig. 7 and Supplementary Figs. 10 and 11.

In the group treated with the vehicle, the last animal was sacrificed by day 42. The treatment with trastuzumab resulted in a delayed tumour growth and the last animal in this group was euthanized at day 70. The median survival of trastuzumab-treated mice was 45 days, which was significantly (*p* < 0.0005, Mantel-Cox test) longer than that of mice treated with vehicle (38 days). The survival time of mice treated with a single injection of [^177^Lu]Lu-PEP49989 or a combination with trastuzumab, were both significantly (*p* < 0.0005) longer than the survival of mice treated with trastuzumab alone and median survival time was not reached in these groups. By the time of termination (day 90), six mice out of seven in the group treated with a single injection of [^177^Lu]Lu-PEP49989 and all eight mice in the group treated with [^177^Lu]Lu-PEP49989/trastuzumab combination were alive. Complete tumour remission was seen in five mice in the group treated with the combination therapy and in one mouse of seven treated with a single injection of [^177^Lu]Lu-PEP49989. In the group treated with two injections of [^177^Lu]Lu-PEP49989, two mice were alive at the study termination, the other five mice had to be sacrificed after the second administration due to a decline in body weight. This resulted in a 45-days median survival of mice in this group, which is significantly shorter (*p* < 0.05) compared with mice treated with single injection of [^177^Lu]Lu-PEP49989 and with its combination with trastuzumab.

The mice tolerated the experimental therapies well, except for the group of mice treated with double injections of [^177^Lu]Lu-PEP49989. No significant difference was observed in the average mice weight between the control group and the groups treated with single radionuclide therapy and/or with trastuzumab (Fig. 7c). However, five animals out of seven in the group treated with double injections of [^177^Lu]Lu-PEP49989 experienced weight loss and had to be euthanized during experimental therapy. Pathology examination revealed minimal-to-mild changes in the liver and a trend to mildly increased scores for proximal tubule single-cell apoptosis of animals treated with [^177^Lu]Lu-PEP49989. Details of toxicity evaluation are presented in the Supplementary data (Supplementary Figs. 12–14, Tables 3 and 4).

## Discussion

Although HER2-targeting antibodies and antibody-drug conjugates provide clinical benefits in metastatic breast cancer, most patients will ultimately experience disease progression and die [34]. Expression of HER2 is not seldom preserved in resistant tumours. This prompts the development of HER2-targeting agents with a mode of action not affected by resistance mechanisms. Previously we have demonstrated that [^177^Lu]Lu-ABY-027 is a promising targeting agent capable of significantly extending the survival of mice bearing HER2-expressing human tumours [24]. However, potential immunogenicity in humans should be minimized for successful clinical translation. To address this, potential T-cell epitopes were removed from ABD035. The deimmunized ABD* variant was tested in a clinical setting as a part of the Affibody-ABD fusion protein izokibep binding to interleukin-17 A for the treatment of psoriasis [35, 36]. The results of that study demonstrated that neutralizing antibodies were not developed during three years of monthly treatment (up to 160 mg Q4W), nor was exposure or pharmacokinetics impacted. These data suggest that immunogenicity was not an issue and support the utility of the deimmunized ABD*. Our previous studies have demonstrated that modification of a few amino acids in Affibody molecule-based constructs can substantially alter their affinity and biodistribution [7], which in principle could cause insufficient radionuclide tumour targeting and unfavourable absorbed dose distribution. A diligent characterization of the new tracer was therefore of high importance to prepare for clinical translation.

PEP49989 was successfully produced with high purity using recombinant protein expression in *E. coli*. This is an advantage in comparison with monoclonal antibodies, which require much more expensive production in mammalian cells. Moreover, we have validated earlier that an Affibody-ABD fusion might be produced by peptide synthesis with a subsequent ligation, thus reducing the cost-of-the goods further [25]. Importantly, the use of a site-specific conjugation of DOTA provides a construct with consistent composition and reproducible targeting properties. PEP49989 demonstrated exact refolding after heat treatment (Supplementary Fig. 2) and specific binding to HER2 in vitro (Fig. 2, Supplementary Fig. 4) and in vivo (Fig. 5). The affinity profile of [^177^Lu]Lu-PEP49989 binding to living HER2-expressing cells was the same as the profile of [^177^Lu]Lu-ABY-027 (Table 3). The cellular processing of both [^177^Lu]Lu-PEP49989 and [^177^Lu]Lu-ABY-027 after binding to cancer cells (Fig. 3) was typical for HER2-binding Affibody molecules, which is characterized by a slow internalization [22, 26]. Affinities of PEP49989 binding to both HSA and MSA (25 and 360 pM, respectively, Table 1) were lower than affinities of ABY-027, but taking into account that the blood concentration of albumin is 0.6–0.7 mM, this should not be critical. Indeed, the blood concentration of [^177^Lu]Lu-PEP49989 did not differ significantly (*p* > 0.05) from the concentration of [^177^Lu]Lu-ABY-027 (Fig. 4) during direct comparison in mice. However, the renal uptake of [^177^Lu]Lu-PEP49989 was slightly but significantly higher. It has to be noted that 20% of cardiac output passes the kidneys. Even if a small fraction of the targeting protein is dissociated, it would be filtered away through glomeruli and reabsorbed in the proximal tubuli of the kidneys.

Modification of the ABD moiety could alter off-target interactions of the fusion protein. This had an impact on the uptake of the construct in kidney where uptake of [^177^Lu]Lu-PEP49989 was slightly higher, and in liver and spleen where uptake of [^177^Lu]Lu-PEP49989 was appreciably lower (Fig. 4). Interestingly, [^177^Lu]Lu-PEP49989 provided significantly (1.3-fold) higher retention of activity in tumours at the late time point than [^177^Lu]Lu-ABY-027 (Fig. 4). Overall, the replacement of ABD035 by the deimmunized ABD* had no negative effect on the targeting properties of [^177^Lu]Lu-PEP49989 (Figs. 4, 5 and 6). The results of the therapy study (Fig. 6) demonstrated that a single injection of [^177^Lu]Lu-PEP49989 was significantly more efficient than treatment with trastuzumab. Analysis of pathology data (Supplementary Figs. 13–14, Tables 3 and 4) suggests that the toxicity to the bone marrow is the dose-limiting organ and not the kidney or liver.

The efficacy of targeted radionuclide therapy can be enhanced by combination with other treatment modalities [1]. Sensitization of cancer cells to radiation by trastuzumab has been demonstrated in vitro [37, 38]. Moreover, clinical data show that trastuzumab treatment increases the effect of external radiation therapy of breast cancer [39, 40]. Since Affibody molecules and trastuzumab bind to different epitopes on the extracellular domain of HER2 [41], they could be used simultaneously without interfering with each other. This study has shown that adding of trastuzumab therapy to a single injection of [^177^Lu]Lu-PEP49989 increased complete remission rate. Due to the different toxicity profiles of radionuclide- and antibody-based therapies, this approach was safer than repeated injections of [^177^Lu]Lu-PEP49989. A reservation needs to be made that the timing of the second injection might not be optimal in this study due to the recovery time needed for the blood cells (Supplementary Fig. 14. This has to be evaluated further.

Further expansion of therapy efficacy using PEP49989 might be expected with the application of emerging promising nuclides, such as ^161^Tb (which co-emits a substantial number of conversion and Auger electrons in addition to β^−^-particles) or alpha-emitting radionuclides ^225^Ac and ^227^Th [42, 43]. The DOTA chelator permits stable labelling of targeting agents with ^225^Ac and ^227^Th, although the chelation kinetics is slow necessitating heating [43, 44]. The use of elevated temperatures is a limitation for application for labelling of monoclonal antibodies. However, PEP49989 refolds with high fidelity after warming up to 90^o^C (Supplementary Fig. 2). Labelling with ^177^Lu at elevated temperatures in this study resulted in a targeting construct with subnanomolar affinity (Tables 1 and 3). Thus, there are several directions for further improvement of radionuclide therapy using PEP49989.

## Conclusion

Incorporation of a deimmunized albumin-binding domain had only a minor impact on cells binding affinity of the Affibody molecule-based construct [^177^Lu]Lu-PEP49989. In the murine model, the novel construct had higher uptake in kidneys but lower uptake in the liver and spleen compared with the parental ABD035-containing [^177^Lu]Lu-ABY-027. The retention of [^177^Lu]Lu-PEP49989 in blood was not compromised and the tumour uptake was higher at the late time point. [^177^Lu]Lu-PEP49989 demonstrated anti-tumour effect in an experimental therapy both alone and in combination with trastuzumab and is a promising candidate for clinical translation.

## Electronic supplementary material

Below is the link to the electronic supplementary material.

Supplementary Material 1

## Data Availability

The datasets generated during and/or analysed during the current study are available from the corresponding author on reasonable request.
