# Supplementary material for: Evaluation of a novel 177Lu-labelled therapeutic Affibody molecule with a deimmunized ABD domain and improved biodistribution profile
Source: Eur J Nucl Med Mol Imaging. 2024 Jul 15;51(13):4038–48. doi: 10.1007/s00259-024-06840-5 (PMC11527907; doi:10.1007/s00259-024-06840-5)
Supplement: Supplementary file 1 — Supplementary Material 1 [file 259_2024_6840_MOESM1_ESM.docx]

**SUPPLEMENTARY MATERIAL**

**Evaluation of a novel ^177^Lu-labelled therapeutic Affibody molecule with a deimmunized ABD domain and improved biodistribution profile**

Yongsheng Liu^1^, Maryam Oroujeni^1,2^, Yunqi Liao^1^, Anzhelika Vorobyeva^1^, Vitalina Bodenko^1^, Anna Orlova^3^, Mark Konijnenberg^4^, Matilda Carlqvist^2^, Elisabet Wahlberg^2^, Annika Loftenius^2^, Fredrik Y Frejd^1,2^, Vladimir Tolmachev^1^

^1^ Department of Immunology, Genetics and Pathology, Uppsala University, 751 85 Uppsala, Sweden

^2^ Affibody AB, 171 65 Solna, Sweden

^3^ Department of Medicinal Chemistry, Uppsala University, 751 23 Uppsala, Sweden;

^4^ Department of Radiology & Nuclear Medicine, Erasmus MC, Rotterdam, The Netherlands

^*^ Correspondence: Vladimir Tolmachev. Email: [vladimir.tolmachev@igp.uu.se](mailto:vladimir.tolmachev@igp.uu.se)

**General**

Most of the chemicals used in the study were purchased from Sigma-Aldrich Sweden AB (Stockholm, Sweden). Buffers used for radiolabelling were prepared using high quality Milli-Q water and purified from metal contaminations using Chelex 100 resin (Bio-Rad Laboratories, USA). No-carrier-added ^177^LuCl_3_ was purchased from Curium Pharma (Stockholm, Sweden). The NAP-5 and PD-10 size-exclusion columns were purchased from Cytiva AB (Uppsala, Sweden). Radioactivity was measured with an automated gamma-spectrometer with a NaI (TI) detector (2480 Wizard, Waltham, MA, USA). Data on in vitro studies and in vivo studies were analyzed by unpaired 2-tailed t-test and ANOVA using GraphPad Prism (version 9.00 for Windows; GraphPad Software LLC, San Diego, CA, USA) to determine significant differences.

**Protein Production & Characterization**

PEP49989 was recombinantly produced in *Escherichia coli*. Electrocompetent cells were transformed with expression plasmid through electroporation. Inoculum media (Tryptic Soy Broth and Yeast extract (Sigma-Aldrich) supplemented with 50 mg/L neomycin (Sigma-Aldrich) was added to a baffled 100 mL shake flask followed by inoculation with the construct from several colonies from the transformation plates and incubation in a shake incubator at 37 °C for 5 hours. Autoinduction expression media (500 mL Overnight Express Instant TB medium (Novagen) supplemented with 50 mg/L neomycin) was prepared in a 2000 mL baffled shake flask and inoculated with 10 mL inoculum. The culture was incubated at 37 °C and 150 rpm overnight (16 hours).

Harvest was performed by centrifugation. The cell pellet was re-suspended in buffer supplemented with 20 mM DTT (Sigma-Aldrich) and 1 µL denarase® (c-LEcta, Leipzig, Germany) per gram pellet before sonication using the Sonics VC-750 Vibra-Cell Sonicator. Clarification was done by centrifugation and the supernatants were filtered (0.45 µm).

Finally, the proteins were purified by affinity chromatography (anti-ABD resin, in-house produced) and reversed phase chromatography (RESOURCE^™^RPC, Cytiva) followed by a buffer exchange step to Chelex^®^ treated 0.2 M ammonium acetate pH 6.0. Thereafter the proteins were conjugated with maleimide-DOTA (Macrocyclics) via a free cysteine for 1 hour at 22°C. The molar ratio was 3.4 DOTA chelators per one Affibody molecule to secure that all proteins were conjugated with the chelator. Excess of free maleimide-DOTA was removed by buffer exchange using PD-10 column (Cytiva). All buffers used during and after the mal-DOTA conjugation step were Chelex^®^ treated to reduce the risk for metal contamination. The endotoxin levels were analysed by Endosafe-PTS (Charles River) and were low < 0.45 EU/mg. ABY-027 was recombinantly produced in E.coli as earlier described [ Tolmachev V, Orlova A, Pehrson R, Galli J, Baastrup B, Andersson K, Sandström M, Rosik D, Carlsson J, Lundqvist H, Wennborg A, Nilsson FY. Radionuclide therapy of HER2-positive microxenografts using a 177Lu-labeled HER2-specific Affibody molecule. Cancer Res. 2007 Mar 15;67(6):2773-82.].

Isoelectric points were determined by isoelectric focusing (Novex™ pH 3-10 IEF Protein Gels, 1.0 mm, ThermoFisher) and was 5.2 for PEP49989 and 8.0 for ABY-027.

The RP-UPLC-MS analysis was done using an Agilent 1290 Infinity UHPLC-system equipped with a single quadrupole and AP-ESI and the analytical column , Waters Acquity UPLC CSH C18(1.7µm, 2.1 x 150mm), with gradient of acetonitrile from 10-60 %B in 0.1% TFA during 24 min at flow rate 0.2 mL/min at 45 °C .Correct molecular mass of the constructs were confirmed.

Circular Dichroism (Jasco J-810 spectropolarimeter, Jasco Scandinavia AB) was used to test reversibility of structure after heating to 90°C.


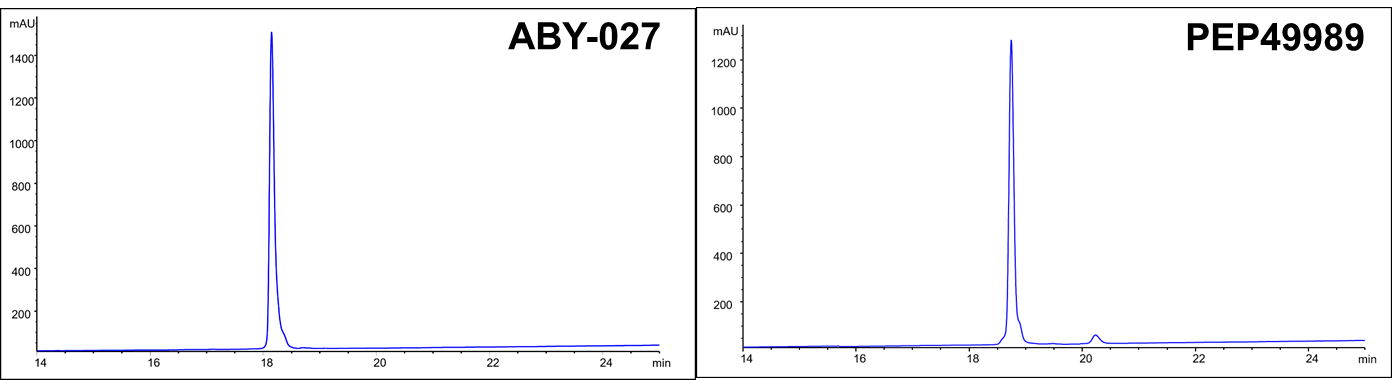


**Supplementary Fig. 1** Reversed-phase HPLC chromatograms of, PEP49989 and ABY-027. UV detector wavelength is at A220nm


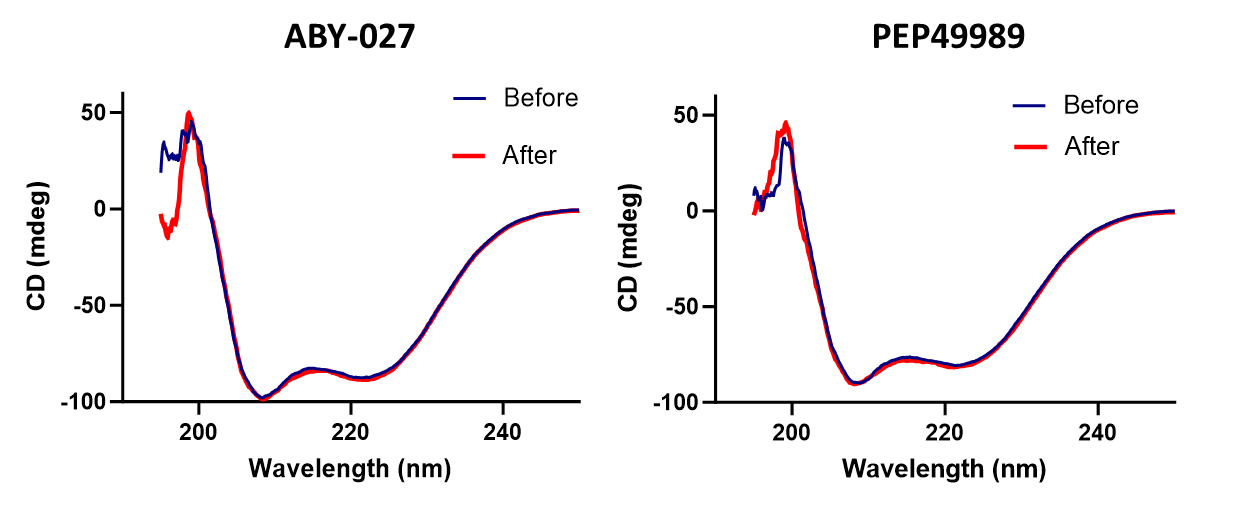


**Supplementary Fig. 2** CD spectra (250-195 nm) before (blue) and after (red) heating (20-90 °C with slope 5 °C/min).

The binding kinetics of ABY-027 and PEP49989 to HER2, human serum albumin (HSA) and mouse serum albumin (MSA) were determined at 25 °C using surface plasmon resonance (Biacore 8K, Cytiva). Binding to HER2 by either ABY-027 or PEP49989 (10 to 0.04 nM) was performed in HBS-EP+ buffer (Cytiva) by immobilization of HER2 to the chip (Series S Sensor Chip CM5, Cytiva) using amine coupling kit type 2 (Cytiva). 10 mM sodium acetate pH 4.5 (Cytiva) was used for immobilization and 25 mM HCl for regeneration.

The binding kinetics to HSA and MSA were determined essentially as described above. However, in these experiments ABY-027 and PEP49989 (10 to 0.04 nM) were injected over immobilized HSA or MSA.

**Radio-HPLC analysis**

To validate iTLC, radio-HPLC of labelled protein was performed. An Elite LaChrom system (Hitachi, VWR, Darmstadt, Germany) consisting of an L-2130 pump, a UV detector (L-2400), and a radiation flow detector (Bioscan, Washington, DC, USA) coupled in series was used. Analysis was performed using an analytical column (Phenomenex, Aschaffenburg,

Germany; Luna® 5 µm C18, 100 Å; 4.6 × 150 mm). HPLC conditions were as follows:

A = 10 mM TFA/H_2_O; B =10 mM TFA/acetonitrile; UV-detection at 214 nm; gradient elution: 0–15 min at 5 to 70% B, 15–17 min at 70 to 95% B, 17–20 min at 5% B; and flow rate was 1.0 mL/min.


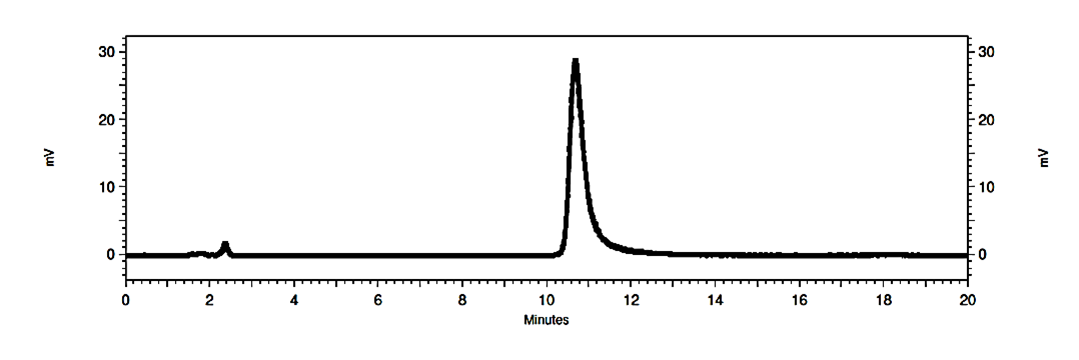
**Supplementary Fig. 3** Radiochromatogram of [^177^Lu]Lu-PEP49989. The peak with the retention time 10.7 min corresponds to cold protein. Radiochemical purity 98.3 % according to radio-HPLC.

**In Vitro Studies**

The binding specificity of [^177^Lu]Lu-PEP49989 to HER2-expressing cells was evaluated by in vitro blocking test using SKOV3 and BT474 cells. Experiments were performed in triplicate. Approximately 1 × 10^6^ cells were seeded per well in 6-well plates the day before the experiment and were maintained in 2 mL complete RPMI 1640 medium at 5% CO_2_ and 37 ℃ environment. For HER2 blocking, cells were incubated with a 1000-fold excess of non-labelled Affibody molecule for 30 min to saturate the receptors. Then, all cells were incubated with [^177^Lu]Lu-PEP49989 (0.5 nM) for 60 min at 37 ℃. After that, the media was collected, the cells were washed with 1 mL PBS, and the fractions were pooled. Thereafter, the cells were detached by incubation with a trypsin-EDTA solution. The radioactivity in cells and media was measured using an automatic gamma counter to calculate the percentage of cell-bound radioactivity. To evaluate the impact of HSA on binding specificity in an additional set of experiments, this protein was added to the complete RPMI 1640 medium to obtain a concentration of 100 nM, and an HSA-containing medium was used for the experiment, performed as described above.

To further confirm that the binding of [^177^Lu]Lu-PEP49989 is HER2-mediated, its binding to HER2-negative cell lines was evaluated in an additional set of experiments. For this purpose, Ramos lymphoma and MDA-MB-468 triple-negative breast cancer cells (both from the American Type Culture Collection) were used. According to the Human Protein Atlas (<https://www.proteinatlas.org/ENSG00000141736-ERBB2/cell+line>, last accessed 3 May 2024), HER2 expression by these cell lines is negligible. Approximately 1 × 10^6^ cells were seeded per well in 6-well plates the day before the experiment and were maintained in 2 mL complete RPMI 1640 medium at 5% CO_2_ and 37 ℃ environment. To ensure the attachment of Ramos cells, the cell culture dishes were treated with polydopamine. The dopamine hydrochloride (Sigma-Aldrich Sweden AB, Stockholm, Sweden) was mixed with 10 mM ris buffer, pH 8.0, to a concentration of 2 mg/mL. Then 600 µL of dopamine solution was added to a 6-well plate and incubated for 3 h at room temperature. The dopamine solution was then removed and the surface was washed twice with water. The Ramos cell suspension in PBS (500 µL containing 1 x 10^6^ cells) was added on the dopamine-coated surface, followed by 60 min incubation at room temperature. After cell attachment, the remaining cell solution was removed, and a cell culture medium containing 1% fetal serum albumin was added to the cells. For HER2 blocking on SKOV3 cells, they were incubated with a 1000-fold excess of non-labelled Affibody molecules for 30 min to saturate the receptors. Then, all cells were incubated with [^177^Lu]Lu-PEP49989 (0.5 nM) for 60 min at 37 ℃ in the presence of human serum albumin. After that, the media was collected, the cells were washed with 1 mL PBS, and the fractions were pooled. Thereafter, the cells were detached by incubation with a trypsin-EDTA solution. A small aliquot (10 µL) was taken to count cell number using TC20 Automated Cell Counter (Bio-Rad Laboratories AB, Solna, Sweden). The radioactivity in cells and media was measured using an automatic gamma counter to calculate the count rate per cell.

The results of this test are presented in Supplementary Fig. 4A. Under the test conditions, [^177^Lu]Lu-PEP49989 retained capacity for a saturable binding to SKOV3 cells. Binding of [^177^Lu]Lu-PEP49989 to HER2-negative cell lines was negligible compared to its binding to HER2-positive cells.

To quantify the number of HER2 receptors per cell in lines with high expression, SKOV3 and BT474 were seeded at a density of 2 x10^5^ cells per well in 400 µL of complete RPMI 1640 medium and were grown overnight. Then, the medium was aspirated, cells were washed with 0.5 mL of cold PBS and 1µg, 200 ng, 40 ng, 8 ng or 1.6 ng of radiolabelled Affibody molecules in 300 µL of cold medium was added to cells. To evaluate an unspecific binding, HER2 receptors in control dishes were blocked by adding 10 µg of non-radiolabelled Affibody molecules 10 min before adding the radiolabelled counterpart. Cells were incubated for 4 h at 4°C on the ice. Then, media was aspirated, cells were washed 3 times with 0.5 mL of cold PBS, detached with 300 µL of trypsin-0.25%EDTA solution and collected for analysis. Cells quantity was measured by TC20 Bio-Rad automated cell counter. The maximum specific binding (B_max_) was determined by “One site-specific binding “ analysis using Prism 10 for Windows, version 10.2.3 (403) (GraphPad Software, LLC (Boston, MA).

The results of this analysis are presented in Supplementary Fig. 4B and 4C. The B_max_ values were 3.96 × 10^-18^ mol/cell for SKOV3 and 3.22 × 10^-18^ mol/cell for BT474 cells. This corresponds to 2.4 × 10^6^ receptors/cell and 1.9 × 10^6^ receptors/cell for SKOV3 and BT474, respectively.


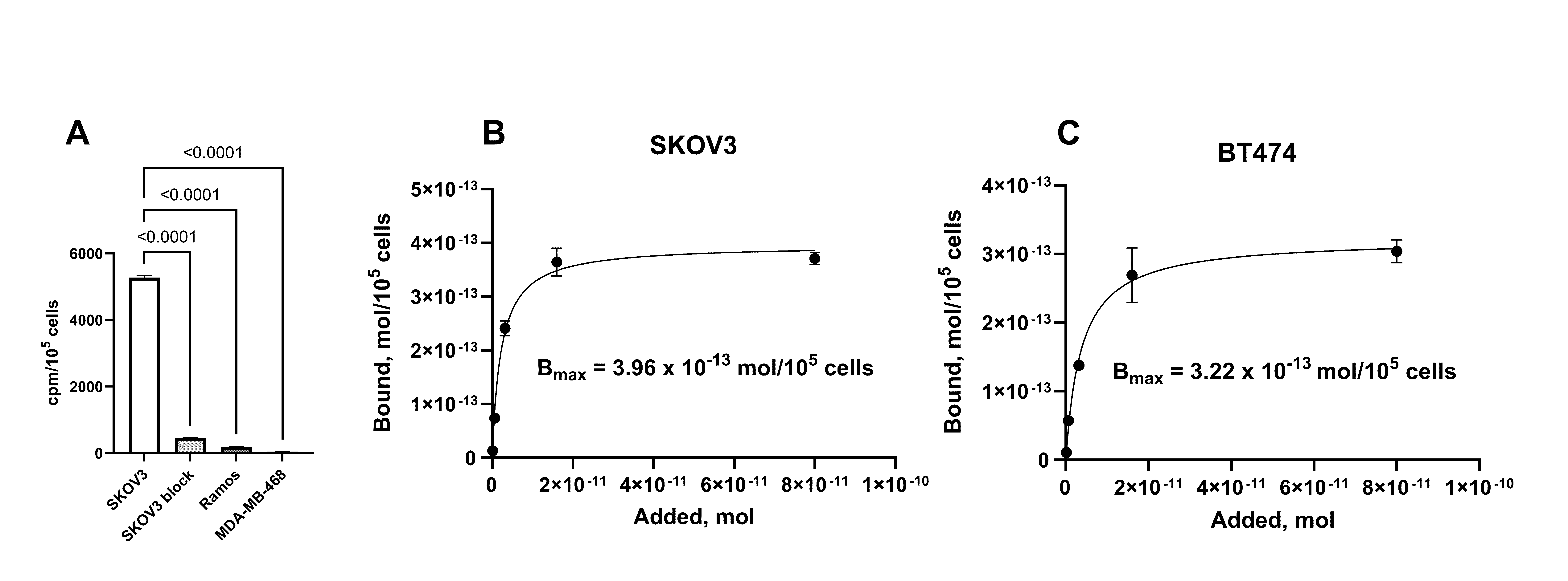


**Supplementary Fig. 4** Characterisation of cell lines**. (**A). Binding of [^177^Lu]Lu-PEP49989 to HER2-negative Ramos lymphoma and MDA-MB-468 triple-negative breast cancer cells. SKOV-3 cells were used as a positive control. Blocked SKOV3 cells were treated with 1000-fold excess of a conjugate. The data are presented as an average value from 3 samples ± SD. P-values from a one-way ANOVA test with Tuckey correction for multiple comparisons are provided. Determination of the maximum specific binding (B_max_) of radiolabelled Affibody molecules to SKOV3 (B) and BT474 (C) cells, respectively.

**Supplementary Fig. 5** Kinetics of binding to living HER2-expressing SKOV3 cells in vitro. LigandTracer sensorgram (A and B) and results of InteractionMap analysis (C and D) of [^177^Lu]Lu-ABY-027 (A and C) and [^177^Lu]Lu-PEP49989 (B and D) binding with the presence of HSA. Binding was measured at three concentrations of 0.25 nM, 0.75 nM and 2.25 nM.

**Cellular processing of [^177^Lu]Lu-PEP49989**

Cellular processing of [^177^Lu]Lu-PEP49989 by HER2-expressing cells were studied in the presence of HSA in SKOV3 and BT474 cell lines using the acid wash method as described in (Liu 2022 Re-188). Approximately 1 × 10^6^ cells were seeded per dish in 3 cm petri dishes. A set of three dishes was used for each data point. Cells were incubated with a 5 nM solution of radiolabelled Affibody molecules at 37 ℃. At 1, 2, 4, 6 and 24 h, the medium was collected from a group of three dishes. The cells were then washed with PBS and treated with 0.2 M glycine buffer containing 4 M urea, pH 2.0, for 5 min on ice. The cells were washed with additional 1 mL glycine buffer, the acidic fractions were collected, and their activity was measured. This activity was considered as membrane bound. After that, cells were incubated with 1 mL of 1 M NaOH at 37 ℃ for 20 min and collected with additional rinsing with 1 mL NaOH. The measured activity in alkaline fractions was considered as an internalized activity.

**In vivo studies**

HER2-positive and HER2-negative xenografts were established by subcutaneous injection of approximately 10^7^ SKOV3 cells or Ramos cells, respectively, in the hind legs of female BALB/C nu/nu mice. Mice were euthanized at pre-determined time points by an intraperitoneal injection of anesthesia, Ketalar-Rompun solution (Ketalar: 10 mg/mL, Rompun: 1 mg/mL). Organs of interest were excised, weighed and their activity was measured. The tissue uptake values were calculated as the percentage of injected dose per gram of the sample (%ID/g).

**Supplementary Fig. 6** Statistical analysis (unpaired t-test) of biodistribution of [^177^Lu]Lu-PEP49989 and [^177^Lu]Lu-ABY-027 in mice bearing SKOV-3 xenografts 48 h after injection . Data are presented as an average (n = 4) value ± SD.

**Supplementary Fig. 7** Statistical analysis (unpaired t-test) of biodistribution of [^177^Lu]Lu-PEP49989 and [^177^Lu]Lu-ABY-027 in mice bearing SKOV-3 xenografts 264 h after injection. Data are presented as an average (n = 4) value ± SD

**Supplementary Fig. 8** Example of biodistribution data fitting.

**Supplementary Table 1.** Dosimetry of [^177^Lu]Lu-PEP49989 in mice.

| Organ | Absorbed dose per injected activity (mGy/MBq) | Absorbed dose after injection of 21 MBq,  Gy |
| --- | --- | --- |
| bone marrow | 59 | 1.24 |
| stomach | 118 | 2.49 |
| total body | 33 | 0.68 |
| pancreas | 116 | 2.43 |
| small intestine | 8 | 0.16 |
| 1iver | 477 | 10.01 |
| spleen | 352 | 7.39 |
| large intestine | 109 | 2.28 |
| kidneys | 482 | 10.13 |
| tumour | 1571 | 33.00 |

For assessment of dosimetry in humans, the murine biodistribution data for [^177^Lu]Lu-PEP49989 were up-scaled using the “percent kg/g method” according to equation 1, as described in

(%IA/organ)_human_=[(%IA/g)_animal_ × (kg_TBweight_)_animal_ × (g_organ_/(kg_TBweight_)_human_] (1)

The organ weight from reference adult female (ICRP publication 89) phantom were used for upscaling. The uptake value was fitted by an exponential function and areas under curves were calculated to determine residence times. OLINDA/EXM 1.0 software was used to estimate absorbed doses.

Estimated absorbed doses after injection of [^177^Lu]Lu-PEP49989 in humans are provided in **Supplementary Table 2**. According to calculations using OLINDA/EXM 1.0, the highest absorbed doses are expected in heart wall, osteogenic cells, small intestine wall, spleen, liver, and kidneys. The expected effective dose is 0.128 mSv/MBq.

**Supplementary Table 2.** Calculated absorbed dose (mGy/MBq) for [^177^Lu]Lu-PEP49989 in humans using OLINDA/EXM 1.0. Literature data for dosimetry (measured in clinics) for two ^177^Lu-labelled monoclonal antibodies are provided for comparison.

| Target | [^177^Lu]Lu-PEP49989 | [^177^Lu]Lu-J591* | [^177^Lu]Lu-lilotomab satetraxetan** |
| --- | --- | --- | --- |
| Adrenals | 0.065 |  | 0.12 (0.10–0.14) |
| Brain | 0.011 |  | 0.10 (0.08–0.13) |
| Breasts | 0.060 |  | 0.10 (0.08–0.12) |
| Gallbladder wall | 0.065 | 0.15 | 0.12 (0.10–0.14) |
| Lower large intest wall | 0.327 |  | 0.11 (0.09–0.13) |
| Small intest wall | 0.252 | 0.12 | 0.11 (0.09–0.13) |
| Stomach wall | 0.074 |  | 0.11 (0.09–0.13) |
| Upper large intest wall | 0.064 | 0.12 | 0.11 (0.09–0.14) |
| Heart wall | 1.410 | 0.95 | 0.11 (0.09–0.13) |
| Kidneys | 0.209 | 1.41 | 0.46 (0.28–0.79) |
| Liver | 0.251 | 2.1 | 0.97 (0.74–1.15) |
| Lungs | 0.163 | 0.75 | 0.11 (0.09–0.13) |
| Muscle | 0.046 | 0.11 | 0.10 (0.08–0.13) |
| Ovaries | 0.063 |  | 0.11 (0.09–0.13) |
| Pancreas | 0.058 | 0.14 | 0.12 (0.1–0.14) |
| Red marrow | 0.121 | 0.32 | 0.94 (0.63–1.28) |
| Osteogenic cells | 0.643 |  | 0.50 (0.31–0.70) |
| Skin | 0.055 |  | 0.10 (0.08–0.12) |
| Spleen | 0.228 | 1.97 | 2.81 (1.54–3.60) |
| Thymus | 0.070 |  | 0.11 (0.09–0.13) |
| Thyroid | 0.058 |  | 0.10 (0.08–0.13) |
| Urinary bladder wall | 0.060 | 0.26 | 0.11 (0.09–0.13) |
| Uterus | 0.062 |  | 0.11 (0.09–0.13) |
| Total body | 0.099 | 0.19 | 0.14 (0.12–0.17) |

*Data from Vallabhajosula S, Kuji I, Hamacher KA, Konishi S, Kostakoglu L, Kothari PA, Milowski MI, Nanus DM, Bander NH, Goldsmith SJ. Pharmacokinetics and biodistribution of 111In- and 177Lu-labelled J591 antibody specific for prostate-specific membrane antigen: prediction of 90Y-J591 radiation dosimetry based on 111In or 177Lu? J Nucl Med. 2005;46:634-41;

** Data from Stokke C, Blakkisrud J, Løndalen A, Dahle J, Martinsen ACT, Holte H, Kolstad A. Pre-dosing with lilotomab prior to therapy with 177Lu-lilotomab satetraxetan significantly increases the ratio of tumour to red marrow absorbed dose in non-Hodgkin lymphoma patients. Eur J Nucl Med Mol Imaging. 2018;45:1233-1241



**Supplementary Fig. 9** Overview of the Therapy experiment.

**Supplementary Fig. 10** Size of SKOV3 xenografts at the day 0 (left) and day 14 (right). The average of tumour volume was significantly lower (p < 0.05, one-way ANOVA with Bonferroni correction for multiple comparisons) in the single or double injection of [^177^Lu]Lu-PEP49989 groups or the combination with trastuzumab from day 14 compared to that in vehicle-treated group.

**Supplementary Fig. 11** The growth of individual tumours in mice treated with (A) vehicle, (B) trastuzumab, (C) single injection (21 MBq) of [^177^Lu]Lu-PEP49989 at day 1, (D) two injections of 21 MBq [^177^Lu]Lu-PEP49989, at days 1 and 29, (E) single injection of [^177^Lu]Lu-PEP49989 and trastuzumab.


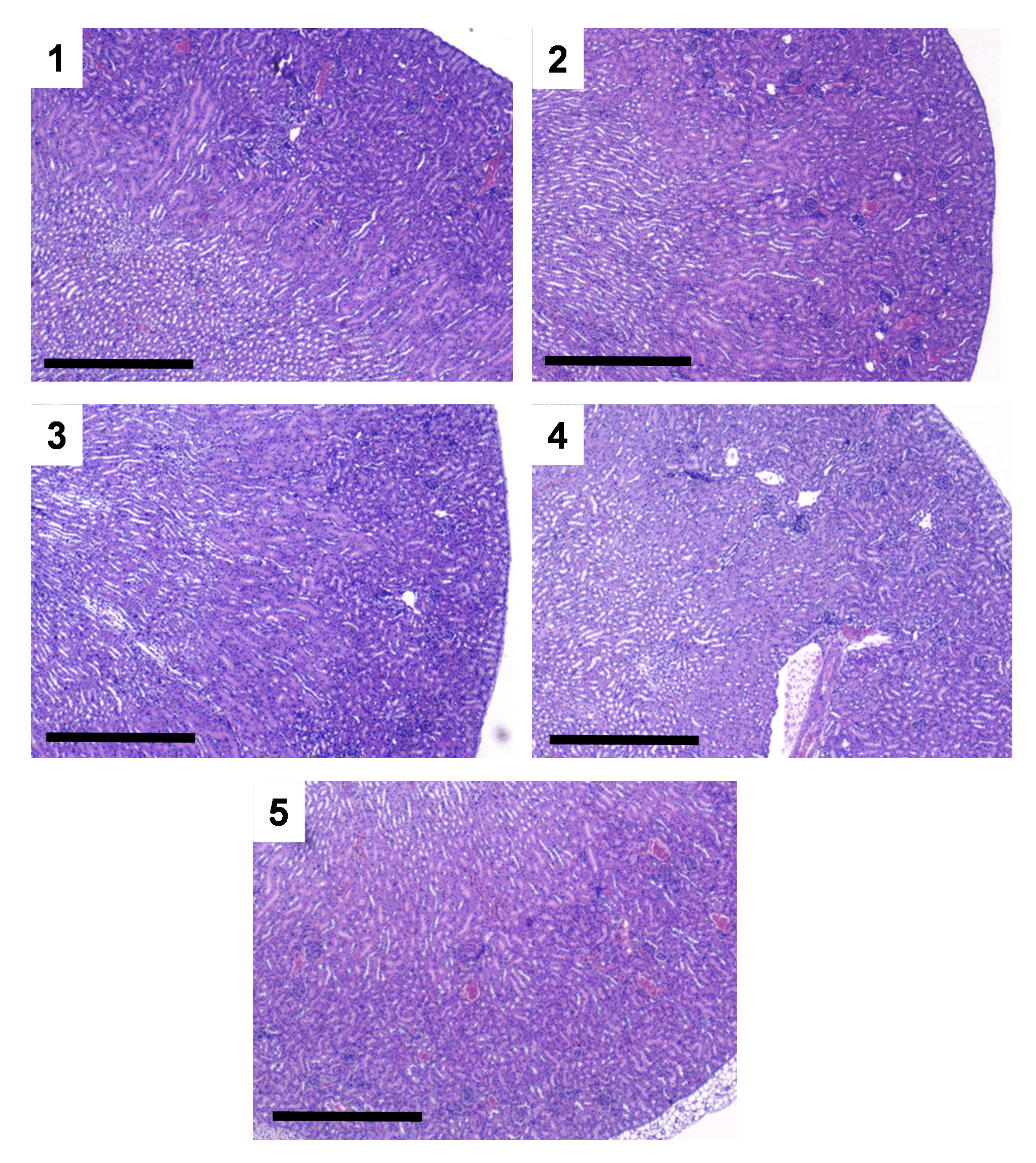


**Supplementary Fig. 12** Histopathological evaluation of formalin-fixed and paraffin-embedded kidneys collected at the endpoint. For histologic evaluation, 4-mm sections were stained with hematoxylin and eosin, Multiplication factor x20, bar corresponds to 100 μm. All sections represent a normal pattern of renal cortex with scattered glomeruli and proximal and distal tubules. 1. Animal from the control group. 2. Animal from the group treated with trastuzumab. 3. Animal from the group treated with a single injection (21 MBq) of [^177^Lu]Lu-PEP49989. 4. Animal from the group treated with two injections (21 MBq) of [^177^Lu]Lu-PEP49989. 5. Animal from the group treated with single injection of [^177^Lu]Lu-PEP49989 and trastuzumab.

**Supplementary Table 3**. Kidney, histopathological changes present in the examined material.

| Experimental treatment ^a^ | Average score per group ^b^ | | | | |
| --- | --- | --- | --- | --- | --- |
|  | Necrosis, single cell: proximal tubules | Desquamated epithelial cells | Tubular casts | Mitotic figures in tubular epithelial cells | Chronic purulent pyelonephritis |
| Control | 0.80±1.10 | 0.00± 0.00 | 0.00± 0.00 | 0.00±0.00 | 0.00±0.00 |
| Trastuzumab | 1.00±0.71 | 0.20±0.45 | 0.00±0.00 | 0.00±0.00 | 0.00±0.00 |
| Single injection of [^177^Lu]Lu-PEP49989 | 1.20±0.45 | 0.80±0.84 | 0.20±0.45 | 0.20±0.45 | 0.80±1.79 |
| Two injections of [^177^Lu]Lu-PEP49989 | 2.00±0.00 | 1.50±0.58 | 0.75±0.50 | 0.25±0.50 | 0.00±0.00 |
| Single injection of [^177^Lu]Lu-PEP49989 and trastuzumab | 1.40±0.89 | 0.80±0.45 | 0.20±0.45 | 0.40±0.89 | 0.00±0.00 |

^a^ Information concerning experimental treatment was communicated to the pathologist after processing, histopathological evaluation and scoring was completed, i.e. the pathologist blinded to treatment during evaluation and scoring

^b^ Scoring of examined tissue was performed according to (Mann et al 2012): 0 = Tissue considered to be normal, under the conditions of the study and considering the age, sex and strain of the animal concerned. Alterations may be present, which, under other circumstances, could be considered deviations from normal. 1 = minimal - the amount of change barely exceeds that which is considered to be within normal limits. 2 = slight - in general, the lesion is easily identified but of limited severity. 3 = moderate, the lesion is prominent, but there is significant potential for increased severity. 4 = severe, the degree of change is as complete as possible (occupies the majority of the organ).

Mann PC, Vahle J, Keenan CM, Baker JF, Bradley AE, Goodman DG, Harada T, Herbert R, Kaufmann W, Kellner R, Nolte T, Rittinghausen S, Tanaka T. International harmonization of toxicologic pathology nomenclature: an overview and review of basic principles. Toxicol Pathol. 2012;40(4 Suppl):7S-13S.





**Supplementary Fig. 13** Histopathological evaluation of formalin-fixed and paraffin-embedded livers collected at the endpoint. For histologic evaluation, 4-mm sections were stained with hematoxylin and eosin, Multiplication factor x100, bar corresponds to 100 μm. All sections represent normal hepatocytes with an occasional cell division. 1. Animal from the control group. 2. Animal from the group treated with trastuzumab. 3. Animal from the group treated with a single injection (21 MBq) of [^177^Lu]Lu-PEP49989. 4. Animal from the group treated with two injections (21 MBq) of [^177^Lu]Lu-PEP49989. 5. Animal from the group treated with single injection of [^177^Lu]Lu-PEP49989 and trastuzumab.

**Supplementary Table 4**. Liver, histopathological changes present in the examined material.

| Experimental treatment ^a^ | Average score per group ^b^ | | | | |
| --- | --- | --- | --- | --- | --- |
|  | Mitotic figures (per 10 HPF in each liver lobe i.e. per 23.7 mm^2^) | Microvesicular fatty change | Single cell necrosis (apoptosis) | Karyocyto-megaly | Anisokaryosis |
| Control | 0.0±0.0 | 0.4±0.5 | 0.0± 0.0 | 0.20±0.45 | 0.20±0.45 |
| Trastuzumab | 0.0±0.0 | 0.0±0.0 | 0.0±0.0 | 0.6±0.6 | 0.6±0.6 |
| Single injection of [^177^Lu]Lu-PEP49989 | 0.6±0.6 | 0.0±0.0 | 0.8±0.5 | 1.60±0.55 | 1.60±0.55 |
| Two injections of [^177^Lu]Lu-PEP49989 | 0.0±0.0 | 1.3±0.6 | 0.0±0.0 | 1.0±0.0 | 1.0±0.0 |
| Single injection of [^177^Lu]Lu-PEP49989 and trastuzumab | 0.4±0.5 | 0.0±0.0 | 0.20±0.45 | 1.20±0.45 | 1.20±0.45 |

^a^ Information concerning experimental treatment was communicated to the pathologist after processing, histopathological evaluation and scoring was completed, i.e. the pathologist blinded to treatment during evaluation and scoring

^b^ Scoring of examined tissue was performed according to (Mann et al 2012): 0 = Tissue considered to be normal, under the conditions of the study and considering the age, sex and strain of the animal concerned. Alterations may be present, which, under other circumstances, could be considered deviations from normal. 1 = minimal - the amount of change barely exceeds that which is considered to be within normal limits. 2 = slight - in general, the lesion is easily identified but of limited severity. 3 = moderate, the lesion is prominent, but there is significant potential for increased severity. 4 = severe, the degree of change is as complete as possible (occupies the majority of the organ).

Mann PC, Vahle J, Keenan CM, Baker JF, Bradley AE, Goodman DG, Harada T, Herbert R, Kaufmann W, Kellner R, Nolte T, Rittinghausen S, Tanaka T. International harmonization of toxicologic pathology nomenclature: an overview and review of basic principles. Toxicol Pathol. 2012;40(4 Suppl):7S-13S.

**Evaluation of hematologic toxicity**

To evaluate hematologic toxicity, an additional experiment was performed. Balb/c nu/nu mice were randomized into 4 groups, with 5 animals in each group. Mice in the control group were intravenously injected with 100 µL of vehicle, 1% BSA in PBS. Mice in three treatment groups were intravenously injected with 10 µg of [^177^Lu]Lu-PEP49989, but with a different activity, 35 MBq, 28 MBq, and 21 MBq. Starting from the 4^th^ day after injection, 20 µL of mouse whole blood was collected from the tail vein into MPA micro-pipettes (Boule Medical AB, Spånga, Sweden) and analysed by Exigo H400 Veterinary haematology analyser (Boule Diagnostics AB, Spånga, Sweden) according to manufacturer’s instruction. Blood samples and mice's body weight were measured twice per week. Mice were euthanized if they lost over 10% of their weight within 1 week, or more than 15% since the study began.

The results of the experiment are presented in Supplementary Fig. 14. Injection of [^177^Lu]Lu-PEP49989 was associated with a significant decrease in cell counts in peripheral blood. The decrease in the weight of animals was also observed after injection of [^177^Lu]Lu-PEP49989. This decrease correlated with the injected activity. Four mice injected with 35 MBq were euthanized 10 days after injection and the last mouse 15 days after injection. All animals injected with 28 MBq reached the humane endpoint 15 days after injection and only mice injected with 21 MBq survived further. For this group of mice, the nadir of platelets count was 15 days after injection, white blood cells 18 days after injection, red blood cells 21 days after injection. Thereafter, the recovery was observed for both body weight and the number of blood cells.

**Supplementary Fig. 14** Body weight and peripheral blood counts of white blood cells (WBC), red blood cells (RBC) and platelets (PLT) after injection of PBS (control) or [^177^Lu]Lu-PEP49989.

SPECT/CT scans were performed for some mice to visualize the expression of HER2 during experimental therapy, using nanoScan SPECT/CT (Mediso Medical Imaging Systems, Budapest, Hungary). On day 79 after treatment initiation, two mice from group treated with a single injection [^177^Lu]Lu-PEP49989 and two from combination treatment group were injected with 5 µg of [^99m^Tc]Tc-ZHER2:41071 Affibody molecule (12.5 MBq), and imaging was carried out at 4 h post-injection (**Supplementary Fig. 15**). In the mice with visible tumours (A and C), growing xenografts were clearly visualized, and the tumour uptake exceeded the uptake in any other organs in these animals. In mice without visible tumour (B and D), the highest accumulation of activity was in kidneys. Only one kidney was observed for mouse B.

**
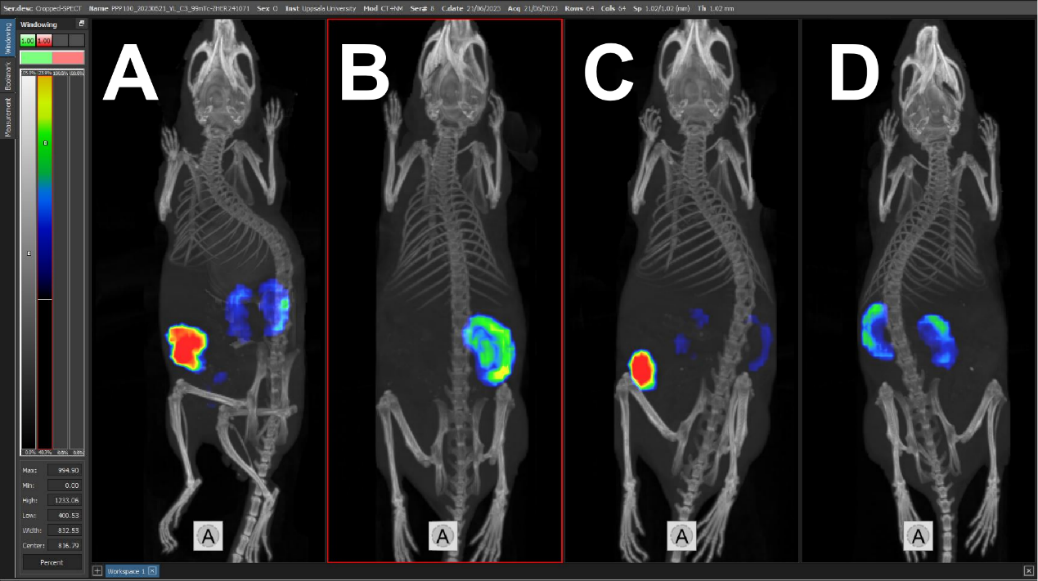
**

**Supplementary Fig. 15** The SPECT/CT imaging using [^99m^Tc]Tc-ZHER2:41071 (maximum intensity projection, sagittal view) of mice after treatment with single [^177^Lu]Lu-PEP49989 treatment (A and B) and in two mice from group treated with combination therapy (C and D). Imaging was performed 4 h after injection of [^99m^Tc]Tc-ZHER2:41071 at day 79 after treatment started.
